# Effect of *Bacillus amyloliquefaciens* and *Bacillus subtilis* on fermentation, dynamics of bacterial community and their functional shifts of whole-plant corn silage

**DOI:** 10.1186/s40104-021-00649-0

**Published:** 2022-01-07

**Authors:** Jie Bai, Marcia Franco, Zitong Ding, Lin Hao, Wencan Ke, Musen Wang, Dongmei Xie, Ziqian Li, Yixin Zhang, Lin Ai, Xusheng Guo

**Affiliations:** 1 State Key Laboratory of Grassland Agro-ecosystems, School of Life Sciences, College of Pastoral Agriculture Science and Technology, Lanzhou University, Lanzhou, 730000 China; 2 Probiotics and Biological Feed Research Centre, Lanzhou University, Lanzhou, 730000 China; 3 Production systems, Natural Resources Institute Finland (Luke), FI-31600 Jokioinen, Finland; 4 School of Food Science and Engineering, Shanxi Agricultural University, Taigu, 030801 China; 5 China Animal Agriculture Association, Beijing, 100044 China

**Keywords:** *Bacillus* silage inoculants, Function prediction, Silage quality, *Zea mays*

## Abstract

**Background:**

*Bacillus amyloliquefaciens* (BA) and *Bacillus subtilis* (BS) are usually used as feed supplements directly or bacterial inoculants in biological feeds for animals. However, few research have reported the effects of BA and BS on fermentation characteristics and bacterial community successions of whole-plant corn silage during ensiling. If the BA and BS inoculants have positive effects on silages, then they could not only improve fermentation characteristics, but also deliver BA or BS viable cells to ruminants, which would play its probiotic effect. Therefore, the objectives of this study were to investigate the effects of BA and BS on the fermentation, chemical characteristics, bacterial community and their metabolic pathway of whole-plant corn silage.

**Results:**

Freshly chopped whole-plant corn was inoculated without or with BA and BS, respectively, and ensiled for 1, 3, 7, 14 and 60 d. Results showed that BA and BS inoculations increased lactic acid concentrations of whole-plant corn silages compared with control, and BA inoculation decreased acetic acid concentrations, whereas BS inoculation decreased fiber contents and increased crude protein (CP) content. Higher water-soluble carbohydrate contents and lower starch contents were observed in BA- and BS-inoculated silages compared with that in control. The decreased CP content and increased non-protein nitrogen content were observed in BA-inoculated silage, which was consistent with the higher amino acid metabolism abundances observed in BA-inoculated silage. In addition, it was noteworthy that BA and BS inoculations increased the metabolism of cofactors and vitamins, and decreased the relative abundances of drug resistance: antimicrobial pathways. We also found that the bacterial metabolism pathways were clearly separated into three clusters based on the ensiling times of whole-plant corn silage in the present study. There were no significant differences in bacterial community compositions among the three groups during ensiling. However, BA and BS inoculations decreased the relative abundances of undesirable bacteria such as *Acetobacter* and *Acinetobacter*.

**Conclusion:**

Our findings suggested that the BS strain was more suitable as silage inoculants than the BA strain in whole-plant corn silage in this study.

## Introduction

Whole-plant corn silage, which makes up over 40% of forage fed to dairy cows [1], has become the main roughage used in ruminants’ diet especially for dairy cattle diets worldwide. Hence, nutrionally and hygiencially high-quality silage is a crucial preprequisite for developing ruminant husbandary. To produce high quality silage, silage inoculants are usually used to promote fermentation process. In the report of Xu et al. [2], silage inoculants were divided into four generations according to their different functions during ensiling. Homolactic bacteria, accelerating the lactic acid fermentation and improving the fermentation quality and nutrients preservation of silage, were identified as the first generation inoculants. Heterolactic bacteria, improving aerobic stability by producing acetic acid and 1, 2-propanediol, were subsequently identified as second generation inoculants. Some strains with special functions, such as feruloyl esterases-producing *Lactobacillus plantarum* [3] or *Pediococcus acidilactici* with high-antioxidant activity [4] that improved the digestibility of fiber or antioxidant capacity in silages, were defined as third generation inoculants. Directly fed microbes, such as *Saccharomyces cerevisiae*, were defined as fourth generation silage inoculants where probiotic microbes delivered direct benefits to the animals through silages. *Bacillus* could also be classified as fourth generation silage inoculants because of its favorable ability to improve animal performance when orally administered [5] and improve fermentation quality and aerobic stability in alfalfa silages [6].

*Bacillus amyloliquefaciens* and *Bacillus subtilis* are usually used as biological control agents to protect plants effectively against plant pathogens [7, 8]. In addition, due to the antimicrobial ability of *B. amyloliquefaciens* and *B. subtilis*, they are used as feed supplements directly or bacterial inoculants in biological feeds for monogastric animals, such as poultry and pigs [9–11]. There are also some studies reported the application of *B. subtilis* in silages. Lara et al. [12] and Bai et al. [6] found that *B. subtilis* used in silages improved fermentation quality and inhibited aerobic spoilage. However, few research have reported the effects of *B. amyloliquefaciens* on fermentation characteristics in silages. Sansinenea and Ortiz [13] reported that *B. amyloliquefaciens* was related to *B. subtilis* and had the potential to produce many antimicrobial compounds. Thus, we speculated that *B. amyloliquefaciens* could also improve fermentation characteristics of silages and deliver *B. amyloliquefaciens* viable cells to ruminants.

Understanding the succession of bacterial community could provide deep insight into the fermentation process underlying silage formation. The bacterial community succession at different phases of ensiling is a dynamic process that varies throughout the fermentation period, and single-molecule real-time (SMRT) sequencing technologies provide useful information on microbial shifts at the species level [14]. In addition, the potential functions of bacterial communities were predicted in some studies, such as Zhang et al. [15] and Bai et al. [16], where they found that the main bacterial functions could explain the material conversion during the composting or ensiling process. To date, the effects of lactic acid bacteria (LAB) inoculants on bacterial community diversity and succession of silages using SMRT sequencing technology have been evaluated in previous studies [16, 17]. LAB, as a commonly used silage inoculants, have been studied a lot in alfalfa silages. However, few researches have reported the effects of *B. amyloliquefaciens* or *B. subtilis* on bacterial community succession and their functional shifts in whole-plant corn silage. Therefore, the objective of this study was to investigate the effects of *B. amyloliquefaciens* or *B. subtilis* on the fermentation characteristics, bacterial community succession and their functional shifts of whole-plant corn silage during ensiling.

## Materials and methods

### Silage preparation

Whole-plant corn (*Zea mays* L. Dajingjiu 3876) was harvested at half milk-line from 4 randomly selected sites in a commercial farm located in Dingxi city, Gansu province, China. The whole-plant corn was chopped into 2 cm size by using a forage cuter (Toyohira Agriculture Machinery, Sapporo, Japan) and then taken into the laboratory immediately. For each of the 4 randomly selected sites, there were 15 piles of forage (1 untreated pile and 2 inoculated piles for each fermentation time of 1, 3, 7, 14 and 60 d). The corn forage piles were treated separately with distilled water (control, CK); *Bacillus amyloliquefaciens* HRH_317_ (BA, 1 × 10^6^ colony-forming unit/g fresh matter, provided by Shanxi Agricultural University, China), which could inhibit fungus by producing antifungal proteins [18]; *Bacillus subtilis* CP7 (BS, 1 × 10^6^ colony-forming unit/g fresh matter, provided by Zhangye Aolin Beier Biological Technology Co., Ltd., China), which had the ability to inhibit gram-negative bacteria, such as *Escherichia* and *Salmonella* by producing antibacterial peptides [6]. The whole-plant corn forage treated with inoculants was then packed into vacuum-sealing polyethylene plastic bags and vacuum-sealed. Each of the groups was ensiled using 4 replicates per group with approximately 300 g of forage per bag. The whole-plant corn silage bags were stored at room temperature (25 ± 2 °C) and sampled after 1, 3, 7, 14 and 60 d of ensiling period.

### Fermentation characteristics and chemical compositions analyses

Twenty g of each fresh and ensiled sample was put in a juice extractor and squeezed with 180 mL distilled water for 30 s, and then filtrated through four layers of cheesecloth. The filtrate pH was measured using a glass electrode pH meter. Determination methods of lactic, acetic, and propionic acid concentrations were referenced by the description of Zhang et al. [4]. Chemical compositions of silages at 60 d of ensiling period were performed as described below. The determination methods of contents of non-protein nitrogen (NPN), ammonia nitrogen (NH_3_-N), and water-soluble carbohydrate (WSC) were referenced by the description of Licitra et al. [19] and Ke et al. [20]. The chemical compositions of silages were presented based on their dry matter (DM) base. The DM content of samples (100 g) was measured by drying them at 65 °C for 72 h, and then ground with a mill (1 mm screen) for nutritional composition analyses [20]. The determination methods of crude protein (CP), neutral detergent fiber (aNDF) and acid detergent fiber (ADF) contents were referenced according to AOAC [21] and van Soest [22]. The starch content of each sample was determined and corrected for free glucose using a total starch determination kit (Bray Bussiness Park, Bray, Co. Wicklow, A98 YV29, Ireland).

### Microbial composition using SMRT analyses

Total genomic DNA of surface bacteria of fresh and ensiled whole-plant corn from the three groups fermented for 1, 3, 7, 14, and 60 d were extracted using a DNA isolation kit (Tiangen, DP302–02, Tiangen, China). Among the four replicates of each treatment, three replicated samples were randomly chosen for DNA extraction. The quality and quantity of extracted DNAs were measured according to the description of Guo et al. [14]. The PCR (Polymerase Chain Reaction) amplification of the bacterial full-length 16S rRNA genes was performed using the forward primer 27F (5′-AGRGTTYGATYMTGGCTCAG-3′) and the reverse primer 1492R (5′-RGYTACCTTGTTACGACTT-3′). Sample-specific 16-bp barcodes were incorporated into the primers for multiplex sequencing. The PCR amplicons were purified with Agencourt AMPure beads (Beckman Coulter, Indianapolis, IN, USA) and quantified using the PicoGreen dsDNA Assay kit (Invitrogen, Carlsbad, CA, USA). The sample was then used to generate a library by using SMRTbell Template Prep Kit 1.0-SPv3, and sequencing was performed using the PacBio platform with DNA/Polymerase Binging Kit 3.0 (PacBio) at Wuhan Frasergen Bioinformatics Co, Ltd. (Wuhan, China).

The rdp_classifier-2.2 software was used to annotate the species of OTU representative sequence basing the database of SILVA 138 firstly. For those OTU representative sequences that annotated to the genus levels but not to the species levels, such as *Lactobacillus*, *Weissella*, *Leuconostoc* and *Pedicoccus*, the blastn (blast-2.11.0+) program based on the best hit method was further used to assign the annotations to the species level by depositing the data in the SILVA 138 library [23, 24]. Before the follow-up statistical analysis, unknown species were filtered at each species level (genus and species), and the abundance of filtered species was classified into others. The communities or species that have significant differences among the three groups were calculated using the linear discriminant analysis effect size analysis (LEfSe). Bacterial function prediction was proof checked from the Kyoto Encyclopedia of Genes and Genomes (KEGG) database using Phylogenetic Investigation of Communities by Reconstruction of Unobserved States (PICRUSt2), which predicts the functional abundance of samples based on the abundance of marker gene sequences in the sample [25].

### Statistical analysis

The experimental protocol had a 3 × 5 factorial design with 3 inoculants and 5 ensiling times. The data for pH and organic acids were analyzed using the general linear model procedure of the Statistical Package for Social Science (SPSS 21.0, SPSS, Inc., Chicago, IL) according to the model:

$$ {Y}_{ij}=\mu +{T}_i+{D}_j+{\left(T\times D\right)}_{ij}+{\varepsilon}_{ij} $$

where *Y*_*ij*_ represents the response variable, μ is the overall mean, *T*_*i*_ is the effect of inoculants, *D*_*j*_ is the effect of ensiling time, *(T × D)*_*ij*_ is the effect of the interaction between the inoculants and ensiling time, and *ε*_*ij*_ is the random residual error. Chemical compositions of 60-d silage samples were analyzed using one-way ANOVA. Tukey’s test was also used for pair-wise mean comparisons. Significance was considered at *P* < 0.05.

## Results

### Chemical compositions of whole-plant corn forage before ensiling

The chemical compositions of fresh whole-plant corn are presented in Table 1. The DM content of the whole-plant corn was 291 g/kg of fresh weight (FW). The starch content was 107 g/kg DM, CP content was 78.6 g/kg DM, αNDF content was 494 g/kg DM, ADF content was 266 g/kg DM, and WSC content was 273 g/kg DM.

**Table 1:** Chemical composition of whole-plant corn forage before ensiling

Items	Whole-plant corn
DM, g/kg FW	291
WSC, g/kg DM	273
Starch, g/kg DM	107
CP, g/kg DM	78.6
αNDF, g/kg DM	494
ADF, g/kg DM	266

*DM*, dry matter; *FW*, fresh weight; *WSC*, water soluble carbohydrates; *CP*, crude protein; *αND*F, neutral detergent fiber assayed with a heat stable amylase and expressed inclusive of residual ash; *ADF*, acid detergent fiber expressed inclusive of residual ash

### Fermentation characteristics and chemical compositions of whole-plant corn silage

Fermentation characteristics and chemical compositions of whole-plant corn silages are listed in Tables 2 and 3, respectively. Overall, the pH of the three groups decreased rapidly and kept stable after 3 d of ensiling. The concentrations of lactic and acetic acids in the three silage groups increased with the extension of the ensiling period (Table 2). The highest lactic acid concentration was observed in BA-inoculated silage, and the lactic acid concentration of BS-inoculated silage was higher than that in control during the entire ensiling period (*P *< 0.001). The acetic acid concentration was higher in BA-inoculated silage compared with control after 1, 3, and 7 d of ensiling, while decreased after 14 and 60 d of ensiling period. The higher acetic acid concentration was in BS-inoculated silage compared with control at the ensiling stages of 1 to 14 d (*P *< 0.001), and no differences were observed after 60 d of ensiling period. Higher lactic acid/acetic acid (LA/AA) ratios were observed in BA- and BS-inoculated silages than that in control after 1, 3, 7, and 14 d of ensiling period, and the lowest LA/AA ratio was observed in BA-inoculated silage after 60 d of ensiling (*P *< 0.001). No propionic acid was detected in all whole-plant corn silages during the ensiling period.

**Table 2:** Fermentation characteristics of the whole-plant corn silage during ensiling

Items	Treatment^**1**^	D^**2**^, d					Mean	SEM^**3**^	***P***-value ^**4**^		
		1	3	7	14	60			T	D	T × D
pH	CK	4.24	4.04	4.04	4.09	3.98	4.08	0.002	< 0.001	< 0.001	< 0.001
	BA	4.23	4.04	4.03	4.00	3.95	4.05				
	BS	4.23	4.04	4.01	4.01	3.98	4.05				
lactic acid, g/kg DM	CK	11.3	22.9	37.8	48.4	63.0	36.9	0.52	< 0.001	< 0.001	< 0.001
	BA	16.5	25.7	48.4	75.7	72.7	47.8				
	BS	16.7	29.3	45.9	54.1	67.9	42.8				
Acetic acid, g/kg DM	CK	4.26	8.57	17.0	25.3	34.6	18.0	0.09	< 0.001	< 0.001	< 0.001
	BA	6.67	9.35	21.0	23.2	29.4	17.9				
	BS	5.73	10.2	21.6	26.4	34.9	19.8				
Lactic acid/Acetic acid ratio	CK	2.55	2.67	2.31	1.93	2.07	2.31	0.009	< 0.001	< 0.001	< 0.001
	BA	2.81	2.75	2.38	1.95	2.03	2.38				
	BS	2.88	2.99	2.37	1.99	2.06	2.46				

^1^*CK*, control, no inoculations; *BA*, silages inoculated with *Bacillus amyloliquefaciens*; *BS*, silages inoculated with *Bacillus subtilis*

^2^*D*, Ensilage time

^3^*SEM*, standard error of the mean

^4^*T*, inoculants treatment; D, fermentation time; *T × D*, the interaction between inoculants treatment and fermentation time

**Table 3:** Chemical compositions of the whole-plant corn silage after 60 d of ensiling

Items^**1**^	Treatments^**2**^			SEM^**3**^	***P***-value
	CK	BA	BS		
DM, g/kg	27.4	28.1	28.1	0.25	0.405
WSC, g/kg DM	159^b^	186^a^	181^a^	4.5	0.002
Starch, g/kg DM	94.3^a^	62.5^b^	75.5^b^	0.51	0.005
CP, g/kg DM	84.0^b^	82.2^c^	85.3^a^	0.46	< 0.001
NPN, g/kg total N	97.3^b^	144.1^a^	92.0^c^	8.29	< 0.001
NH_*3*_*-N*, g/kg total N	68.2	61.5	68.4	1.55	0.097
αNDF, g/kg DM	481^a^	478^a^	468^b^	0.2	< 0.001
ADF, g/kg DM	252^a^	248^a^	235^b^	0.3	< 0.001

^a-c^ Means within a row without a common superscript letter differ

^1^*DM*, dry matter; *WSC*, water soluble carbohydrates; *CP*, crude protein; NPN, non-protein nitrogen; *NH*_3_*-N*, ammonia nitrogen; *αNDF*, neutral detergent fiber assayed with a heat stable amylase and expressed inclusive of residual ash; *ADF*, acid detergent fiber expressed inclusive of residual ash

^2^*CK*, control, no inoculations; *BA*, silages inoculated with *Bacillus amyloliquefaciens*;* BS*, silages inoculated with *Bacillus subtilis*

^3^*SEM*, standard error of the mean

The BA and BS inoculations increased WSC contents and decreased starch contents when compared with control (*P *< 0.05, Table 3). The highest CP content was observed in BS-inoculated silage, followed by control, and then in BA-inoculated silage (*P *< 0.001). On the other hand, the highest NPN content was in BA-inoculated silage, followed by control, and then in BS-inoculated silage (*P *< 0.001). However, no differences were observed in NH_3_-N contents among the three groups. The aNDF and ADF contents were lower in BS-inoculated silage compared with control and BA-inoculated silage (*P *< 0.05).

### Bacterial community composition and diversity in whole-plant corn silage

**Fig. 1:**
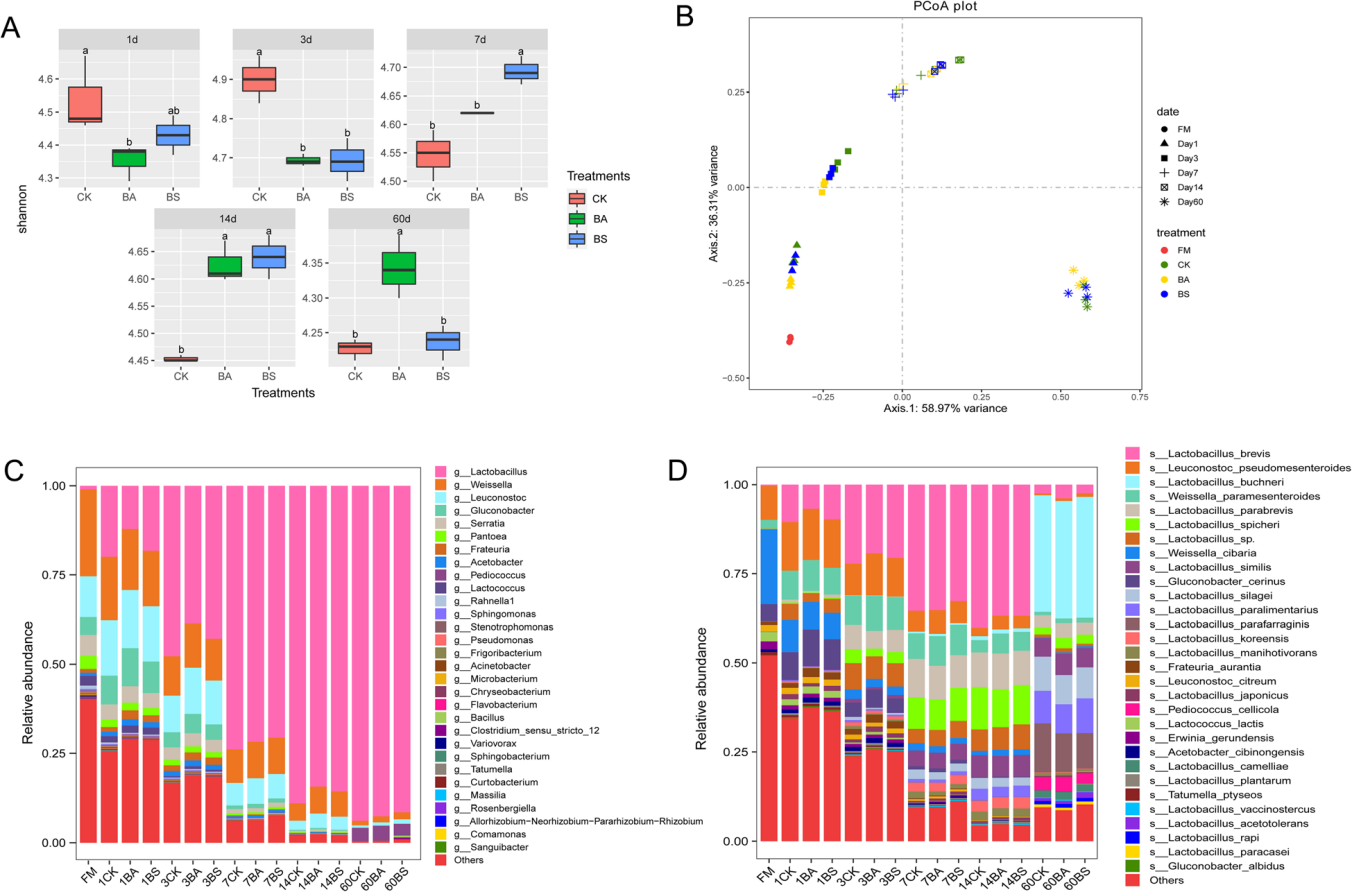
Bacterial community diversities and compositions in whole-plant corn silage during ensiling. CK, Control (samples without inoculants); BA, samples inoculated with *Bacillus amyloliquefaciens*; BS, samples inoculated with *Bacillus subtilis*. Arabic number indicating days of ensiling. **A**. The variations in community alpha-diversities (Shannon index). **B**. The community dissimilarities in different groups and fermentation times, calculated using Principal Coordinates Analysis (PCoA). **C**. Relative abundances of whole-plant corn silage bacterial genera across different groups and fermentation times. **D**. Relative abundances of whole-plant corn silage bacterial species across different groups and fermentation times

**Fig. 2:**
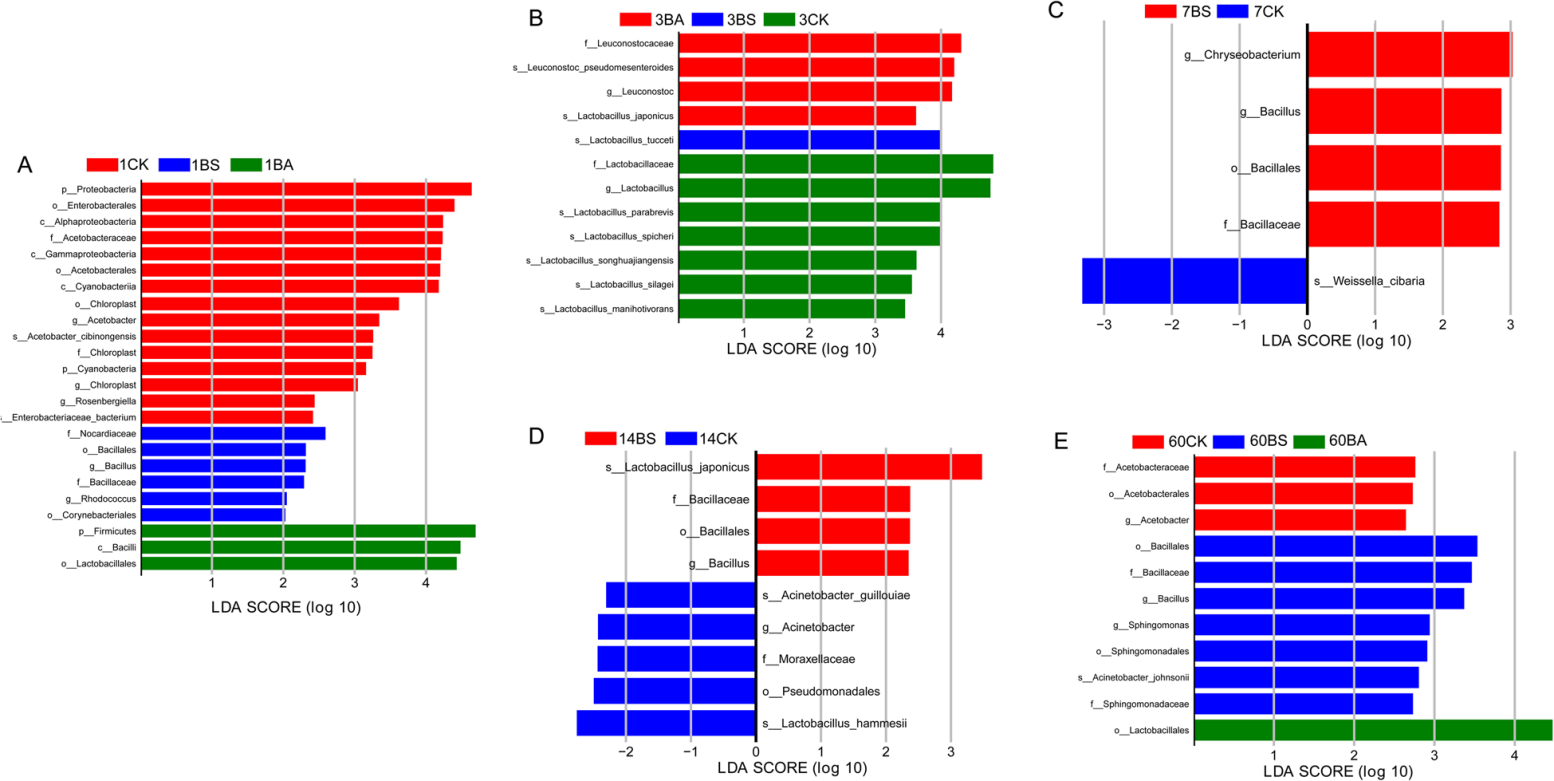
Identification of the communities or species that have significant differences among the three groups. CK, Control (samples without inoculants); BA, samples inoculated with *Bacillus amyloliquefaciens*; BS, samples inoculated with *Bacillus subtilis*. Arabic number indicating days of ensiling. **A.** after 1 d of ensiling; **B.** after 3 d of ensiling; **C.** after 7 d of ensiling; **D.** after 14 d of ensiling; **E.** after 60 d of ensiling

Bacterial diversity, bacterial community compositions and differences are shown in Fig. 1 and 2, respectively. The alpha diversities were lower in BA- and BS-inoculated silages compared with control after 1 and 3 d of ensiling period. However, the alpha diversity was lower in control after 7, 14, and 60 d of ensiling period (Fig. 1A). According to the beta diversity (PCoA, Principal Coordinates Analysis), significant differences and regular changes in bacterial community successions at the different fermentation stages were observed. Nevertheless, the bacterial communities in the three groups were not clearly separated (Fig. 1B). The bacterial community dynamics of whole-plant corn silages at the genus and species level are shown in Fig. 1C and D, respectively. Before ensiling, the main epiphytic bacteria at the genus level were *Weissella* and *Leuconostoc*, and *Gluconobacter*, *Serratia*, *Pantoea*, and *Lactococcus* also had higher relative abundance. The main epiphytic bacterial species are *Weissella cibaria* (20.92%) and *Leuconostoc pseudomesenteroides* (9.51%). After ensiling, the bacterial community compositions of the three silage groups were similar, and obvious regular changes were observed with the extension of ensiling. The relative abundance of *Lactobacillus* increased rapidly, and *Weissella*, *Leuconostoc*, and *Gluconobacter* decreased during the fermentation process. After 60 d of ensiling, the compositions of dominant bacteria at genus levels became simple that *Lactobacillus* was the main genus, and *Lactococcus* appeared in the three silage groups, and *Lactococcus* was higher in the BA inoculated silage. In addition, the species of *Lactobacillus* varied during the ensiling. After 60 d of ensiling, the bacterial community compositions at the species level were completely changed. The dominant *Lactobacillus* changed from *L. brevis* (> 30%) after 7 and 14 d of ensiling to *L. buchneri* (> 30%) after 60 d of ensiling. However, no differences were observed on the main bacterial community compositions between BA- and BS-inoculated silages and control.

Due to the low relative abundance of *Bacillus*, we only annotated *Bacillus* at the genus levels, but did not annotated *B. amyloliquefaciens* and *B. subtilis* at the species levels in this study. Therefore, we performed a statistical analysis on the relative abundance of *Bacillus* (Table 4). We found that *Bacillus* was not detected in the fresh sample and control. The higher relative abundance of *Bacillus* was observed in BS-inoculated silage than that in BA-inoculated silage. In addition, the relative abundance of *Bacillus* in BS-inoculated silage was increased with the extension of ensiling time, while the relative abundance of *Bacillus* kept stable during the entire ensiling period.

**Table 4:** Relative abundance of *Bacillus* during ensiling

D^**1**^, d	Treatments^**2**^			SEM^**3**^	***P***-value
	CK	BA	BS		
1	0^c^	0.0104^b^	0.057^a^	0.0088	<0.001
3	0^c^	0.0137^ab^	0.0259^a^	0.0046	0.033
7	0^b^	0.0104^b^	0.0587^a^	0.0093	<0.001
14	0^b^	0.0104^b^	0.0466^a^	0.0075	0.002
60	0^b^	0.0104^b^	0.4664^a^	0.0771	<0.001

^a-c^ Means within a row without a common superscript letter differ

^1^*D*, Ensilage time

^2^*CK*, control, no inoculations; *BA*, silages inoculated with *Bacillus amyloliquefaciens*; *BS*, silages inoculated with *Bacillus subtilis*

^3^*SEM*, standard error of the mean

Then we explored the differences in bacterial communities among the three groups using the LEfSe analysis (Fig. 2). After 1 d of ensiling, the *Chloroplast*, and *Acetobacter* were higher in control, while *Bacillus* and *Rhodococcus* were higher in BS-inoculated silage, and *Lactobacillales* and *Bacilli* were high in BA-inoculated silage. After 3 d of ensiling, the significantly different bacteria were all lactic acid bacteria (LAB) among the three groups, *Leuconostoc* was in control, *Lactobacillus* was in BA-inoculated silage. *Bacillus* was higher in BS-inoculated silages after 7, 14, and 60 d of ensiling period. *Acinetobacter* and *Acetobacter* were higher in control than that in BA- and BS-inoculated silages after 14 and 60 d of ensiling, respectively. *Lactobacillales* was still higher in BA-inoculated silage than that in the other two groups after 60 d of ensiling period.

### Bacterial metabolic functions and enzyme shifts during ensiling

**Fig. 3:**
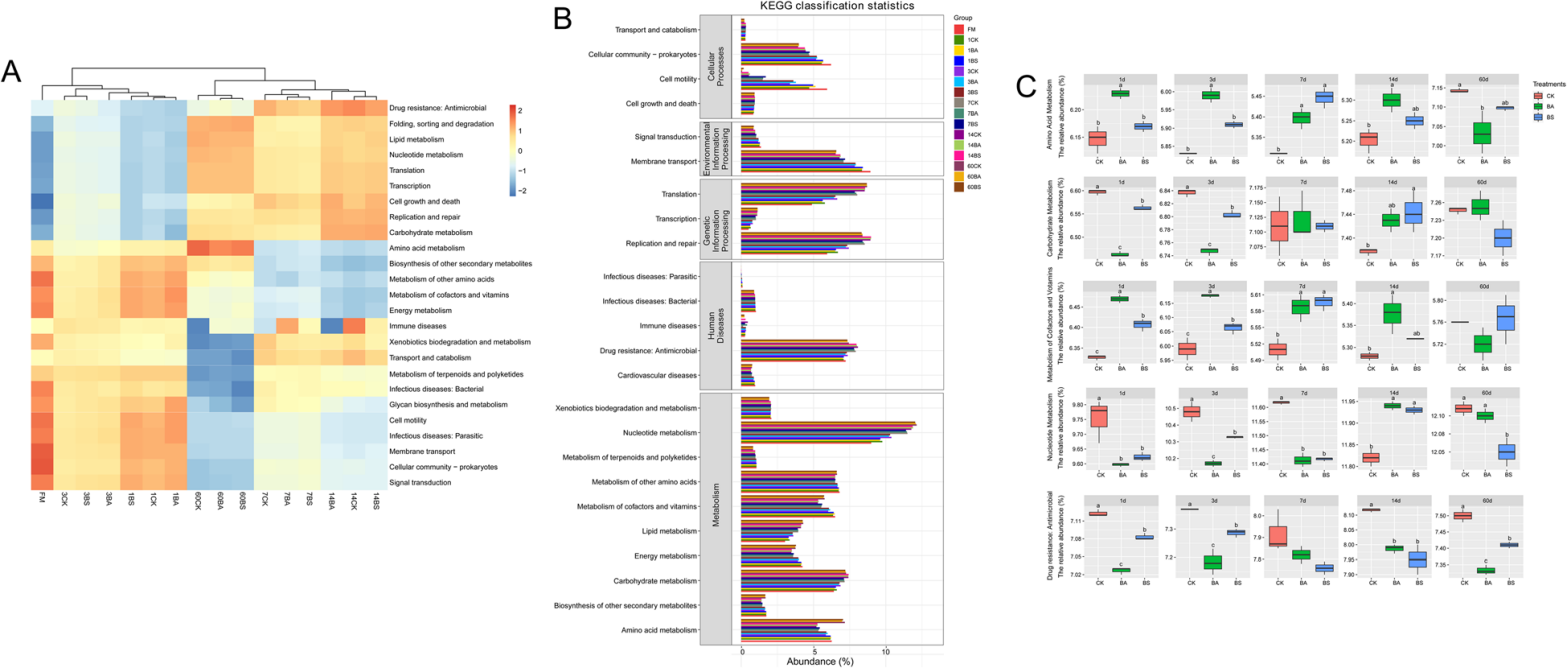
Bacterial alterations that contribute to functional shifts after fermentation in different groups. CK, Control (samples without inoculants); BA, samples inoculated with *Bacillus amyloliquefaciens*; BS, samples inoculated with *Bacillus subtilis*. Arabic number indicating days of ensiling. Summary of functional shifts predicted using Phylogenetic Investigation of Communities by Reconstruction of Unobserved States (PICRUSt2). For each KEGG pathways, the second level of the predicted functional shift is shown with respect to the different groups and fermentation processes. **A**. Clustering heat map of functional abundance (top 25 abundant functions) of whole-plant corn silage. The color corresponding to the middle heat map represents the Z value obtained after the relative abundance of the function is normalized. The closer the color is to red, the higher the abundance. **B**. Level 2 KEGG orthologue functional predictions explained by PICRUSt2. **C**. Significant differences of the 5 functional pathways among the three groups with the same silage period at *P *< 0.05

**Fig. 4:**
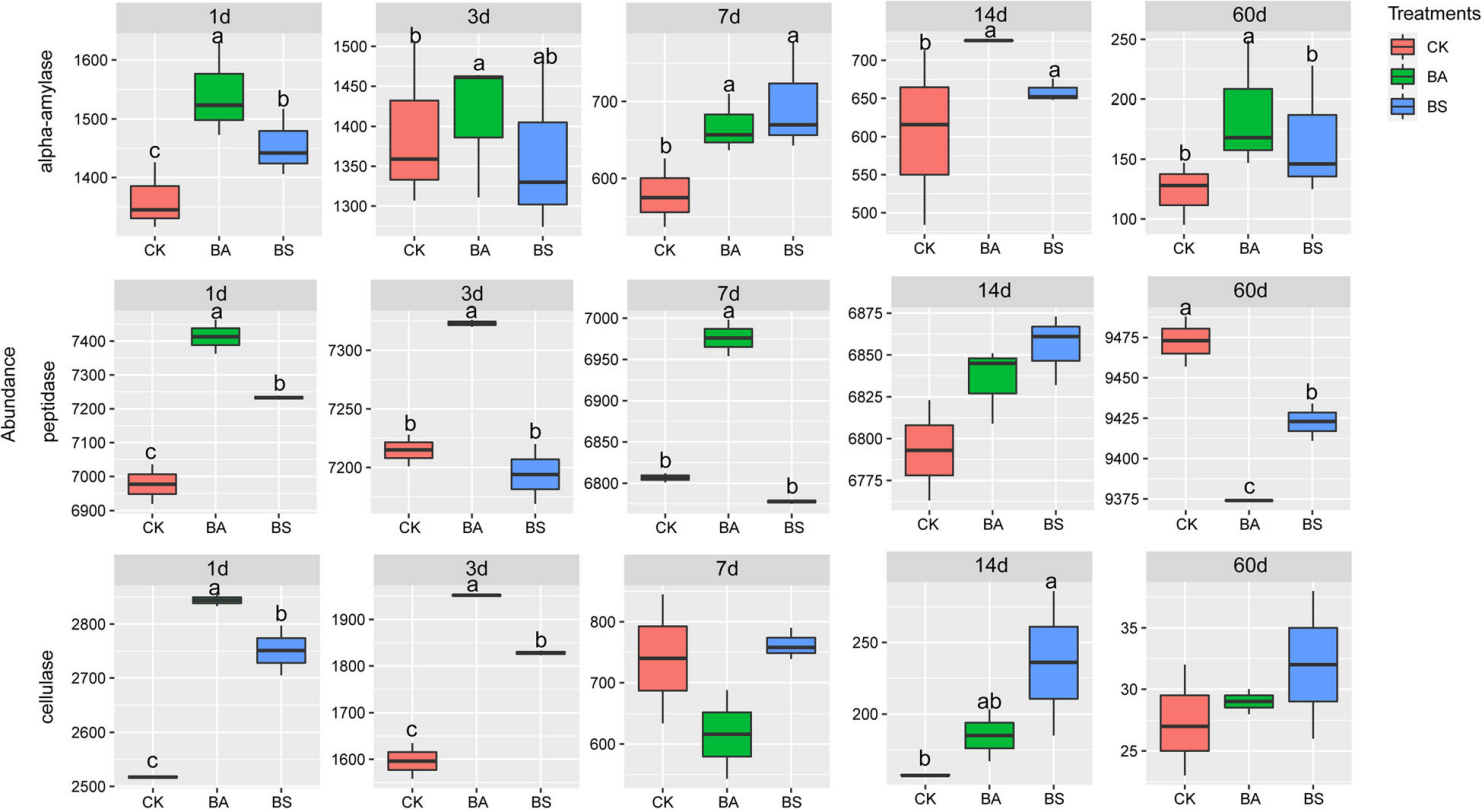
Bacterial alterations that contribute to enzyme shifts after fermentation in different groups. CK, Control (samples without inoculants); BA, samples inoculated with *Bacillus amyloliquefaciens*; BS, samples inoculated with *Bacillus subtilis*. Arabic number indicating days of ensiling. Summary of enzyme shifts predicted using Phylogenetic Investigation of Communities by Reconstruction of Unobserved States (PICRUSt2). Significant differences of the 3 enzymes among the three groups with the same silage period at *P *< 0.05

The potential functions and enzyme of bacterial communities of the three groups at different ensiling stages were predicted by the PICRUSt2 software (Fig. 3 and 4). The heat-map of the top 25 predicted functional abundance is shown in Fig. 3A. During the ensiling, the ensiling process was clustered into three categories according to the changes of bacterial functions, namely silages ensiled for 1 and 3 d, 7 and 14 d, and 60 d. The majority of predicted functions explained with KEGG pathways were grouped into cellular processes (4 pathways), environmental information processing (2 pathways), genetic information processing (3 pathways), human diseases (5 pathways), and metabolism (10 pathways) in the three silage groups (Fig. 3B). Among them, carbohydrate metabolism, amino acid metabolism, nucleotide metabolism, and metabolism of cofactors and vitamins were chosen as main metabolic pathways due to their relative abundance accounted for more than 5% during the whole ensiling process. The relative abundances of nucleotide metabolism and carbohydrate metabolism of the three silage groups increased with the extension of ensiling period. The relative abundances of amino acid metabolism and metabolism of cofactors and vitamins decreased at the stages of 1 to 14 d of ensiling period, while increased after 60 of ensiling period. In addition, the drug resistance: antimicrobial pathway was the main human disease pathway associated with the silage bacteria. Then the 5 function abundances were analyzed by statistical analysis (Fig. 3C). The higher relative abundances of amino acid and cofactors and vitamins metabolism were observed in BA-inoculated silages compared with control after 1, 3, 7, and 14 d of ensiling period, while decreased after 60 d of ensiling period. The relative abundances of carbohydrate metabolism were higher in control compared with BA- and BS-inoculated silages after 1 and 3 d of ensiling period, while the relative abundance of carbohydrate metabolism increased in the BA- and BS-inoculated silages after 7 d of ensiling period. The abundances of nucleotide metabolism were higher in control than that in BA- and BS- inoculated silages during the whole ensiling process except after 14 d of ensiling period. It is noteworthy that the lowest abundance of drug resistance: antimicrobial pathway was in the BA inoculated silage, followed by the BS inoculated silage.

EC:3.2.1.1 (α-amylase), EC:3.2.1.4 (cellulase) and EC:3.4.21.107 (peptidase) that are closely related to silage fermentation were chosen in enzyme classification (EC) database (Fig. 4). Overall, abundances of α-amylase and cellulase of whole-plant corn silage with or without inoculations decreased with the extension of ensiling time (Fig. 4A and C), while peptidase abundance decreased during ensiling period from 7 to 14 d and then increased after 60 d of ensiling (Fig. 4B). The abundance of α-amylase was higher in BA-inoculated silage when compared with control during the entire ensiling periods. Higher abundance of peptidase was observed in BA-inoculated silages compared with that in control and BS-inoculated silages during the ensiling periods from 1 to 7 d, while decreased in BA-inoculated silage after 60 d of ensiling. The abundance of cellulase was higher in BA- and BS-inoculated silage than that in control after 1, 3, and 14 d of ensiling, while no differences of cellulase abundance were observed between control and inoculated silages after 60 d of ensiling.

## Discussion

The highest lactic acid concentration was observed in BA-inoculated silage, and the lactic acid concentration of BS-inoculated silage was slightly higher compared with control during ensiling period, which might be explained that BA- and BS-inoculations accelerated lactic acid fermentation during ensiling. According to Guo et al. [14], applying *Lactobacillus buchneri* accelerated the lactic acid-producing bacteria *L. plantarum* growth to some extent. In addition, it is interesting that the increase in lactic acid concentrations did not cause a decrease in pH of BA- and BS-inoculated silages. Little differences were observed in pH between the control and inoculated silages in value in the present study. In our previous study, higher concentrations of lactic acid and NH_3_-N, and higher pH were all in *Enterococcus faecalis* inoculated silage. Thus, we speculated that NH_3_-N neutralized the acid to prevent the decrease of the pH value [16]. However, no differences in NH_3_-N contents were observed among the three silage groups, so the possibility that NH_3_-N neutralized acid was ruled out in this study. Therefore, it is hard to explain the phenomenon that the differences of the lactic acid contents were observed while no differences were found in pH between the control and inoculated silages. The acetic acid concentration was higher in BS-inoculated silage compared with control during the ensiling period from 1 to 14 d, but no difference was observed between control and BS-inoculated silage after 60 d of ensiling. Similar results also reported in our previous study that BS-inoculation increased the lactic acid concentrations but have no effect on the acetic acid concentrations of alfalfa silage when compared with the control after 60 d of ensiling [6]. The BA-inoculation increased acetic acid concentration during the ensiling period from 1 to 7 d when compared with control, while decreased from the ensiling periods from 14 to 60 d. It might be indicated that the BA strain used in this study accelerated lactic acid fermentation, while inhibited acetic acid fermentation of whole-plant corn silage. Kung et al. [26] reported that the LA/AA ratio was commonly used as a qualitative indicator of fermentation, and great fermentation of silages have LA/AA ratio of about 2.5 to 3.0. In the present study, the LA/AA ratio in the three groups decreased to less than 2.5 during 7 to 60 d of ensiling processes. In addition, Muck and Kung [27] pointed that a slightly higher LA/AA ratio was observed in silages inoculated with homolactic acid inoculants, which might be explained by the only lactic acid produced by homolactic LAB. We found that slightly higher ratios of LA/AA were observed in BA- and BS-inoculated silages during the stages of 1 to 14 d of ensiling, indicating that the BA and BS inoculations accelerated the homolactic acid bacteria in silages when the fermentation initiated.

The WSC is the critical component for LAB fermentation to produce high-quality silages [28]. Higher WSC contents were observed in BA- and BS-inoculated silages after 60 d of ensiling period in this study. Meanwhile, we found that the contents of starch were lower in BA- and BS-inoculated silages. Kleinschmit and Kung [29] reported that *L. buchneri* inoculation reduced WSC contents of grass and small-grain silages compared with the silage without inoculations by a meta-analysis study. Our previous study also showed that *L. buchneri* and BS inoculations decreased WSC contents of alfalfa silages compared with control [6]. Considering the differences of WSC and starch contents between the control and inoculated silages in the present study, the degradation of starch is probably the result of enzyme mechanisms by the *Bacillus* inoculation. To confirm this hypothesis, we used the hydrolysis transparent circle method to verify and found that a transparent circle was produced when BA was inoculated on a plate containing soluble starch. Therefore, the BA strain produced amylase to degrade the starch to WSC during ensiling in the present study. According to the previous studies, starch and hemicelluloses could be degraded into simple carbohydrates by microbial and plant enzymes under acidic conditions [30, 31]. However, starch which generally accounts for 60–80% of ruminant diets, is the main energy source for the organism and rumen microbes [32]. The massive degradation of starch in whole-plant corn silage is an energy loss for ruminants, even though higher residual WSC degraded by starch in the silage is nutritionally desirable for ruminants. Thus, from the perspective of starch preservation in corn silage, the strains of BA and BS were not suitable for use as whole-plant corn silage inoculants. However, when feeding corn grains to ruminants, the degradation rate of corn starch in the rumen is only 62% [33]. On the other hand, the fermentation of excessive starch in the rumen will cause changes in the rumen environment, affecting the utilization of other nutrients, and even metabolic diseases. More and more researches have focused on the solid-state fermented feed by *Bacillus* in livestock production [10, 11]. Therefore, the BA and BS inoculations could be used as inoculants of solid-fermented corn to investigate the effect of rumen starch degradation in further study. The highest CP content was in BS-inoculated silage, followed by control, and the last was the BA-inoculated silage. On the contrary, the highest NPN content was in BA-inoculated silage, followed by control, and the last was the BS-inoculated silage. The result supported in our previous study indicated that the BS inoculation inhibited the proteolysis in alfalfa silage [6]. However, the BA inoculation hydrolyzed CP into large amounts of NPN in the present study. We knew that NPN in the silage was less efficiently synthesized by rumen microbial nitrogen than true protein [34]. Although the inoculation of BA increased the loss of true protein, the proteases metabolized by BA might help increasing starch digestibility. According to Kotarski et al. [35], the exposed endosperm could not be fully digested as the existence of a starch-protein matrix formed in the chemical bonding of zein protein with starch granules. Therefore, the zein protein might be hydrolyzed by protease to release starch granules, which facilitated the hydrolysis of starch by amylase, which might be explained by the lowest starch content in BA-inoculated silage. Then we confirmed that the BA strain produced protease to degrade protein by using the hydrolysis transparent circle method that a transparent circle was produced when BA was inoculated on a plate containing casein. The reduced contents of aNDF and ADF were in BS-inoculated silage when compared with control after 60 d of ensiling, which was consistent with our previous study that BS inoculation decreased aNDF and ADF contents in alfalfa silage [6]. However, the BA inoculation did not have the ability to degrade aNDF and ADF.

No difference was observed in bacterial diversity of whole-plant corn silages with the extension of ensiling time according to the Shannon index in the present study. However, Xu et al. [17] showed that the decreased alpha diversity was observed when the compositions of dominant LAB became relatively simple during ensiling. In the present study, complete changes were observed in the compositions of the dominant bacterial species during the ensiling of the corn silage, but the compositions did not become simple. This might be the explanation of alpha diversity kept stable during the entire ensiling process. The bacterial Shannon index (alpha diversity) was higher in control compared with that of BA- and BS-inoculated silages during the ensiling period from 1 to 3 d, while the Shannon index decreased in control compared with BA- and BS-inoculated silages from 7 to 60 d of ensiling. It was probably due to the antibacterial substance produced by the strains BA and BS during the early ensiling period that inhibited the growth of undesirable microorganisms, and consequently decreased bacterial diversity. The beta diversity analysis revealed a significant temporal and regular succession pattern among the ensiling stages, identifying that ensiling time as a factor which shaped the differences of whole-plant corn microorganism during the ensiling period. In addition, the bacteria of silages fermented for 7 and 14 d clustered together, indicating that there was little change in bacterial diversity of whole-plant corn silage during ensiling period from 7 to 14 d. However, the bacterial diversity was indistinguishable among control and silages inoculated with BA or BS, which was in accordance with the report of Xu et al. [17] that *L. plantarum* and *L. buchneri* inoculations did not clearly separate bacterial diversity of whole-plant corn silage compared with control.

To future reveal the bacterial community compositions of whole-plant corn silage inoculated with the strains of BA and BS, we evaluated bacterial communities at genus and species level. The relative abundances of *Lactobacillus* increased gradually during the entire ensiling process. *Lactobacillus*, rod-shaped LAB, plays a predominant role in lactic acid fermentation in silages [36]. Guan et al. [37] reported that *Lactobacillus* dominated in the early stages of corn ensiled without silage inoculants. However, *Lactobacillus* dominated in the entire stage of ensiling, especially during 7 to 60 d of ensiling in this study, which was in accordance with the report of Ni et al. [38] that *Lactobacillus* played crucial roles in pH decrease at the later stage of ensiling. The compositions of the dominant bacteria in the three groups were similar, but the relative abundances had certain degrees of differences. The relative abundances of *Weissella*, *Leuconostoc*, *Gluconobacter*, and *Serratia* were also higher in whole-plant corn silages after 1 d of ensiling except *Lactobacillus*. The relative abundances of the four genera decreased with the extension of ensiling until they were completely replaced by *Lactobacillus* and *Pediococcus* after 60 d of ensiling period. In addition, species of *Lactobacillus* also underwent a complete change that *L. brevis*, *L. parabrevis*, *L. spicheri*, and *L. similis* at the stages of 1 to 14 d of ensiling completely changed to *L. buchneri*, *L. silagei*, *L. paralimentarius*, and *L. parafarraginis* after 60 d of ensiling. Furthermore, we found that BA and BS inoculations increased the relative abundances of *Leuconostoc pseudomesenteroides* and *Weissella paramesenteroides* of whole-plant corn silage compared with control, which might explain the increased lactic acid concentrations in BA- and BS-inoculated silages. *Leuconostoc* and *Weissella* as lactic-acid-producing cocci initiated lactic fermentation during the stages of early ensiling, while they had lower tolerance to low pH than *Lactobacillus* [38, 39]. Additionally, we found that the BS inoculation promoted the growth of *Weissella* in alfalfa silage in our previous study [6]. The strain *Gluconobacter cerinus* and their role in silage fermentation were not reported in previous studies. The genus *Gluconobacter*, which belongs to the group of acetic acid bacteria, is the most important producer of wine spoilage which is legally defined by volatile acidity, largely composed of acetic acid [40, 41]. Therefore, the increased acetic acid concentration in BA-inoculated silage during the ensiling period from 1 to 7 d might be due to the higher relative abundance of *G. cerinus*. The acetic acid concentrations of BA-inoculated silage decreased from 14 to 60 d of ensiling when the growth of the aerobic bacteria *Gluconobacter* was inhibited by the anaerobic environment [40]. The BA and BS inoculations, unlike the *Lactobacillus* inoculation, had little effect on the bacterial community of whole-plant corn silage. Xu et al. [2] reported that the *Saccharomyces cerevisiae* inoculation, a kind of facultative aerobic fungus, had little effect on the bacterial community composition of whole-plant corn during ensiling. While according to Xu et al. [17], *L. plantarum* or *L. buchneri* inoculations resulted in significant differences in bacterial community composition as well as their succession when compared with control in ensiled corn. It might indicate that *Bacillus* and *S. cerevisiae* as unconventional silage inoculations, had a weak competition with the epiphytic microorganisms of whole-plant corn raw materials, especially with the progress of anaerobic ensiling process, they were not enough to change the bacterial community compositions of corn silage.

In order to explore the dynamics of BA and BS additives in the whole-plant silage during ensiling, we performed statistical analysis on the relative abundance of *Bacillus*. Because of we only annotated *Bacillus* at the genus levels, but did not annotated *B. amyloliquefaciens* and *B. subtilis* at the species levels in this study. We found that the relative abundance of *Bacillus* was zero in the fresh sample and control during the entire ensiling, indicating that *Bacillus* was not present in the whole-plant corn silage that is naturally fermented in the present study. Therefore, the *Bacillus* detected in BA- and BS-inoculated silages should be the added ones. In addition, we found that the higher relative abundance of *Bacillus* was observed in BS-inoculated silage than that in BA-inoculated silage. Meanwhile, the relative abundance of *Bacillus* in BS-inoculated silage was increased with the extension of ensiling time. The result indicated that as facultative aerobic bacteria, the *B. subtilis* strain had higher activity and adaptability in whole-plant corn silage than the *B. amyloliquefaciens* strain in the present study.

To further explore the differences of bacterial community between control and BA- and BS-inoculated silages, we used LEfSe analysis which is a method that coupled standard tests for statistical significance with additional tests encoding biological consistency and effect relevance [42]. *Bacillus* was the most differentially abundant bacteria in the BS-inoculated whole-plant corn silage during the entire ensiling procedure except for 3 d of ensiling. The same result was obtained in our previous study that *Bacillus* was significantly higher in BS-inoculated alfalfa silage than that in control after 60 d of ensiling by LEfSe analysis [6]. It was inferred that *Bacillus* could also grow in silages with anaerobic fermentation. However, *Bacillus* did not increase significantly in BA-inoculated silage. It does not mean that *Bacillus* was not present in BA-inoculated silage. In addition, *Lactobacillales* was significantly higher in BA-inoculated silage than that in control and BS-inoculated silage by LEfSe analysis after 1 and 60 d of ensiling, and *Leuconostoc* was the most differentially abundant bacteria in BA-inoculated silage after 3 d of ensiling, which might be explained by the higher lactic acid concentrations in BA-inoculated silage. *Acetobacter* and *Acinetobacter*, undesirable bacteria in silages, were the most differentially abundant bacteria in control during ensiling, indicating that BA and BS inoculations could inhibit the growth of undesirable bacteria of silage.

The ensiling process is mediated by microbial metabolic pathways to transform metabolites or degrade substrates. We can assess the effect of bacterial communities on the changes in the metabolic pathways during ensiling by predicting bacterial functions. Therefore, the KEGG pathway database with PICRUSt2 was used to predict the bacterial community functions of whole-plant corn silages in this study. We found that the silage fermentation process was clustered into three groups according to the changes of bacterial functions, which could be used to define stages of silage fermentation as early (1 to 3 d of ensiling), mid (7 to 14 d of ensiling), and late (60 d of ensiling) periods. We generally focused on the metabolism of carbohydrates, nucleotide, energy, amino acid and cofactors and vitamins (the main metabolic pathways in silages), which were related to the changes of fermentation and chemical characteristics [16, 17]. In the present study, carbohydrate and nucleotide metabolism of the three silage groups increased with the extension of ensiling. According to Bai et al. [16], the relative abundances of total LAB in the microbial community affected the abundances of carbohydrate metabolism pathway. The increased total LAB abundance was also observed in silages with higher carbohydrate metabolism pathway abundances the present study. According to the report of Kilstrup et al. [43], most of metabolic reactions are related to either bacterial utilization of nucleotides or their regulation by metabolites. Silage fermentation was dominated by LAB, therefore, increases in the total LAB abundance might lead to the enhancement of nucleotide metabolism. The higher relative abundances of nucleotide metabolism were observed in control than that in BA- and BS-inoculated silages during the entire ensiling periods except 14 d of ensiling, which might be due to the slightly higher relative abundance of total dominant LAB in the control. Metabolism of amino acid and cofactors and vitamins decreased during the phases from 1 to 14 d of ensiling period while increased after 60 d of ensiling period. It was hard to explain the reason for this phenomenon. The higher relative abundances of metabolism of cofactors and vitamins were observed in BA- and BS-inoculated silages. In our previous study, the *Enterococcus faecalis* inoculation increased relative abundance of metabolism of cofactors and vitamins in alfalfa silage [16]. It might be inferred that some strains of *Bacillus* could accelerate vitamin production or produce vitamins directly during ensiling. In addition, the amino acid metabolism increased in BA-inoculated silage compare to control during the phases from 1 to 14 d of ensiling, which was in accordance with the decreased CP content and increased NPN content in BA-inoculated silage. That might be the result of protein degradation by the proteases produced by the BA strain.

The abuse of antibiotic results a global growth of multidrug-resistant bacteria, which becomes one of the greatest threats to human health. [44]. In nature, antibiotic-resistance genes exist in bacterial and fungal [45]. Therefore, we chose the drug resistance: antimicrobial pathway to explore the changes of this pathway during ensiling process and the differences among the three groups. The relative abundances of the drug resistance: antimicrobial pathway increased during the 1 to 14 d phase of the ensiling period, while decreased after 60 d of ensiling period. Meanwhile, the highest relative abundances of this pathway were observed in control during the entire ensiling process, and the lowest relative abundances were in BA-inoculated silage, followed by BS-inoculated silage. The antimicrobial drug resistances are mostly found in the undesirable bacteria, such as *Salmonella* and *Escherichia coli* [46, 47]. It might be due to the antibacterial substance produced by BA and BS inoculations that inhibit the growth of undesirable and harmful bacteria and thus reduced the resistance genes.

In order to further confirm the effects of BA and BS inoculants on the chemical characteristics of whole-plant corn silages, EC numbers of α-amylase, peptidase and cellulase were chosen according to PICRUSt2 in this study. We found that the highest abundance of α-amylase was observed in BA-inoculated silage during the entire ensiling period, which was consistent with the lowest content of starch in BA-inoculated silage after 60 d of ensiling. The lowest abundance of α-amylase was in accordance with the highest content starch in control. The result indicated that the BA strain in this study produced α-amylase to degrade starch during ensiling of whole-plant corn silage. In addition, the BA inoculation increased the abundance of peptidase during the early ensiling periods, while the peptidase abundance became the lowest in BA-inoculated silage after 60 d of ensiling. The lowest CP content in BA-inoculated silage might be due to the stronger activity of the facultative aerobe BA strain in the early stage of ensiling, which produced more peptidase in the optimal growth environment. The lower abundance of peptidase was observed in BS-inoculated silage compared with BA-inoculated silage, which was consistent with the higher CP content and lower NPN content in BS-inoculated silage. The changes of cellulase abundance of the three silage groups could not explain the differences in aNDF and ADF contents. We found that the BS inoculation decreased aNDF and ADF contents of whole-plant corn silage compared with control and BA-inoculated silage. However, the cellulase abundance was higher in BA-inoculated silage than that in control and BS-inoculated silage after 1 and 3 d of ensiling, and no difference on the cellulase abundance was observed between BA- and BS-inoculated silages during the ensiling period from 7 to 60 d. Therefore, the decrease in fiber content in BS-inoculated silage might not be related to the abundance the effect of cellulase.

## Conclusions

The BA and BS inoculations had a little effect on bacterial community compositions. However, BA and BS inoculations significantly decreased the relative abundance of *Acetobacter* and *Acinetobacter* according to LEfSe analysis. Higher abundances of *Bacillus* and *Lactobacillales* were observed in BS- and BA-inoculated silage, respectively. Additionally, the whole-plant corn ensiling process could be clustered into three stages through changes in metabolism pathways, which could be a new approach to silage research. We also found that BA inoculation increased the relative abundances of amino acid metabolism, which was consistent with the increased NPN content and the decreased CP content in BA-inoculated silage. Meanwhile, the decreased starch content and increased WSC content were observed in BA-inoculated silage after 60 d of ensiling period. Due to the special digestion and absorption mechanism of ruminants, the degradation of protein and starch by BA might not be suitable for whole-plant corn silage. However, the BS inoculation increased the CP content and reduced the contents of NPN, aNDF, and ADF when compare with control. It is noteworthy that the metabolism of cofactors and vitamins increased in BA- and BS-inoculated silages; meanwhile, the drug resistance: antimicrobial pathway decreased during the ensiling.

## Data Availability

Raw sequencing files and associated metadata have been deposited in NCBI’s Sequence Read Archive (SRR14832366-SRR14832413), https://www.ncbi.nlm.nih.gov/sra.

## Abbreviations

BA: *Bacillus amyloliquefaciens*
BS: *Bacillus subtilis*
SMRT: Single-molecule real-time sequencing technology
LAB: Lactic acid bacteria
DM: Dry matter
FW: Ffresh weight
CK: Control
NPN: Non-protein nitrogen
NH_3_-N: Ammonia nitrogen
CP: Crude protein
αNDF: Neutral detergent fiber
ADF: Acid detergent fiber
WSC: Water-soluble carbohydrate
LA/AA: Lactic acid/Acetic acid
PCR: Polymerase Chain Reaction
PCoA: Principal Coordinates Analysis
LEfSe: The linear discriminant analysis effect size analysis
PICRUSt2: Phylogenetic investigation of communities by reconstruction of unobserved states
KEGG: Kyoto encyclopedia of genes and genomes
SPSS: Statistical package for social science
